# Impact of Insertion Speed, Depth, and Robotic Assistance on Cochlear Implant Insertion Forces and Intracochlear Pressure: A Scoping Review

**DOI:** 10.3390/s24113307

**Published:** 2024-05-22

**Authors:** Filip Hrnčiřík, Leo Nagy, Hannah L. Grimes, Haissan Iftikhar, Jameel Muzaffar, Manohar Bance

**Affiliations:** 1 Cambridge Hearing Group, Cambridge CB2 7EF, UK; fh371@cantab.ac.uk (F.H.);; 2 Department of Clinical Neurosciences, University of Cambridge, Cambridge CB2 0QQ, UK; 3 Clinical School, University of Cambridge, Cambridge CB2 0QQ, UK; 4 Department of Otolaryngology, University Hospitals Birmingham, Birmingham B15 2TT, UK

**Keywords:** cochlear implantation, insertion forces, intracochlear pressure, robotic assistance, hearing preservation, cochlear trauma, insertion depth, insertion speed, insertion approach, electrode design, angular depth

## Abstract

Cochlear implants are crucial for addressing severe-to-profound hearing loss, with the success of the procedure requiring careful electrode placement. This scoping review synthesizes the findings from 125 studies examining the factors influencing insertion forces (IFs) and intracochlear pressure (IP), which are crucial for optimizing implantation techniques and enhancing patient outcomes. The review highlights the impact of variables, including insertion depth, speed, and the use of robotic assistance on IFs and IP. Results indicate that higher insertion speeds generally increase IFs and IP in artificial models, a pattern not consistently observed in cadaveric studies due to variations in methodology and sample size. The study also explores the observed minimal impact of robotic assistance on reducing IFs compared to manual methods. Importantly, this review underscores the need for a standardized approach in cochlear implant research to address inconsistencies and improve clinical practices aimed at preserving hearing during implantation.

## 1. Introduction

The World Health Organization reports that 466 million people worldwide are affected by hearing impairment, which is the most common sensory deficit. The loss of hearing can have a dramatic effect on a person’s quality of life, leading to social stigmatization, isolation, psychological issues, loss of career opportunities, difficulty in relationships and communication, and a higher incidence of depression [1,2,3], dementia [4,5,6], and overall mortality [7,8].

Cochlear implants (CIs) have revolutionized the treatment of severe to profound hearing loss by electrically stimulating the cochlear nerve and bypassing normal hearing mechanisms. Despite their benefits, CIs have limitations in terms of their clinical effectiveness and the number of people who can use them. One of the challenges of CI insertion is the mechanical trauma it generates, which can result in intracochlear tissue damage and an ongoing inflammatory response that can harm residual acoustic hearing [9,10,11]. Preservation of this hearing is important as it can enhance the benefits of combined electro-acoustic stimulation and expand the eligibility of patients for CIs. Several factors may contribute to the occurrence of cochlear trauma during implantation, including the surgical approach, electrode design, and the interaction between these factors. Two main surgical approaches are utilized for cochlear implant insertion: the RW approach and the cochleostomy (CO) approach. Additionally, various designs of cochlear implant electrodes, such as lateral wall (LW) and perimodiolar (PM) variations, can influence the risk of intracochlear trauma during surgery. Understanding the relationship between these factors and cochlear trauma is essential to refining surgical techniques and electrode designs, ultimately leading to improved patient outcomes. The Eshraghi et al. scale serves as an important tool for categorizing trauma levels in cochlear implantation procedures [12]. This scale enables researchers to systematically evaluate the occurrence and severity of trauma and its potential implications for patient outcomes.

To investigate factors such as insertion speed, insertion depth, electrode design, surgical approach and angular depth, and the use of semi-automated or fully automated insertion techniques which influence insertion force (IF) and intracochlear pressure (IP) changes, researchers have employed physical artificial models (e.g., scala tympani or combined-scalae cochlea models fabricated from plastic or similar materials) [11,13,14,15,16,17,18,19,20,21,22,23] as well as computational simulations [24] in their studies. These models provide a controlled environment for observing and measuring the IFs and transient IP changes that arise during CI insertion. However, as artificial models do not normally contain flexible membranes (basilar and Reissner’s) separating the individual scalae, the findings in these models should be accepted with caution [25]. It is important to consider data from cadaveric specimens and also from histologic examinations in order to correlate insertion trauma location with the rise of IFs and IP [9,10,11,26]. To truly understand the impact of CI insertion on residual hearing preservation, it is crucial to consider both artificial models and cadaveric specimens.

A number of parameters have been examined in an effort to reduce mechanical trauma during the implantation process. Researchers have explored using lubricants [16,27,28,29,30] and optimizing insertion speed [11,24]. Lubricants function by lowering the friction between the implant and the cochlear wall during insertion, possibly minimizing IFs, reducing the potential for tissue damage, and, in theory, preserving residual acoustic hearing. Furthermore, optimizing insertion speed might help to mitigate transient IP changes, which may be important for both preserving hearing and not damaging the vestibular (balance) structures [31].

This review aims to provide a comprehensive overview of the current understanding of CI’s IFs and IP and their potential impact on the preservation of residual hearing. An analysis of the existing literature on the measurements of IFs and IP in artificial cochlea models and cadavers, the impact of insertion speed, depth/angle of insertion, electrode design, surgical approach, and the use of robotic assistance during implantation is performed. Lastly, the review evaluates the collected literature concerning cochlear trauma associated with varying surgical methods and types of cochlear implants.

## 2. Materials and Methods

The study was prospectively registered in the PROSPERO database of reviews for human (CRD42021246926) and animal studies (CRD42021247348).

### 2.1. Study Criteria

#### Inclusion Criteria

The types of studies included any experimental study describing IFs in cochlear implantation. This includes case–control, case series, randomized controlled trials, and animal studies (live, explant, in vitro). All animal models (live, explant, in vitro; all species, all sexes) were implanted with a cochlear implant. All studies (live and cadaveric) assessed the insertion trauma or force resulting from cochlear implantation. Cochlear implantation—primary outcome assessed was insertion force measurements (mN) obtained in artificial, cadaveric, and animal models for all brands of cochlear implants with respect to different conditions, such as

- Audiometry measures of hearing preservation;
- Evoked potentials in response to auditory stimuli;
- Hydrostatic pressures measured in the inner ear during insertion;
- Variation in cochlear size;
- Variation in cochlear shape;
- Speed of insertion (if measured);
- Shape of implant tip (if recorded).

### 2.2. Exclusion Criteria

The types of studies excluded review articles, though reference lists were searched, case reports, and studies with insufficient data.

### 2.3. Search Strategy

The search was conducted on 3 April 2021 through the following databases: MEDLINE, Embase, Cochrane, Scopus, and Web of Science. The searches were re-run and confirmed to be up to date on 23 April 2023. The search strategy for MEDLINE was used as a template on which further strategies were developed for the different listed databases. The literature referenced by identified publications was reviewed to screen for further appropriate studies. The search strategy was as follows:

1. Cochlear Implants/or Cochlear Implantation/
2. (“cochlear implant*” or “cochlear device*” or “cochlear electrode*” or “cochlear array*”).tiabkw.
3. “insert*”.tiabkw
4. (“force” or “impact” or “speed” or “pressure”).tiabkw
5. (“insert*” adj2 (“force” or “impact” or “speed” or “pressure”)).tiabkw
6. 1 or 2
7. 5 and 6
8. (“insert*” adj5 (“force*” or “impact*” or “speed*” or “pressure*”)).tiabkw
9. 6 and 8

The MeSH terms used on MEDLINE (Northfield, IL, USA) were ‘Cochlear Implants’ and ‘Cochlear Implantation’.

### 2.4. Study Selection

**Figure 1:**
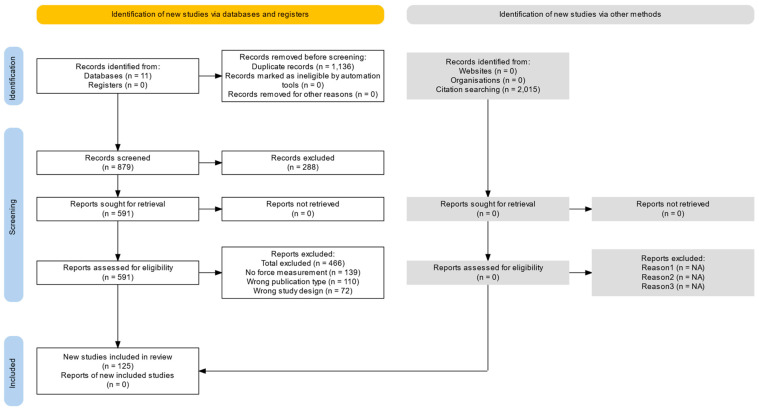
PRISMA flowsheet showing number of search results. The figure was generated using the PRISMA Flow Diagram tool [32].

Two authors independently screened the titles and abstracts of all search results generated by the strategy. The full texts were considered by both authors independently with respect to the inclusion and exclusion criteria. The study identification process is shown in the PRISMA flow chart (Figure 1).

### 2.5. Data Extraction and Management

A standardized spreadsheet was used to guide data extraction from our included publications. Data were extracted from text, tables, and diagrams within the included publications in the following domains:

1. Reference details;
2. Study design;
3. Intervention details;
4. Outcome measurements.

### 2.6. Outcome Measures

The primary outcome measure was force during cochlear implant insertion. The secondary outcome measures included hearing threshold levels, auditory evoked potentials, and histological damage to cochlea.

### 2.7. Statistical Processing and Meta-Analysis

Statistical analyses were performed using MATLAB (MathWorks). An unweighted two-sample *t*-test was employed when the number of studies in each group was unequal, while a weighted *t*-test was utilized when the groups had an equal number of data points. The significance level for the data was set at *p* < 0.05. To obtain data at specific insertion depths from diagrams, WebPlotDigitizer V5 [33] was employed.

## 3. Results

### 3.1. Effect of Insertion Speed Including Human- and Robotic-Assisted Approaches

**Figure 2:**
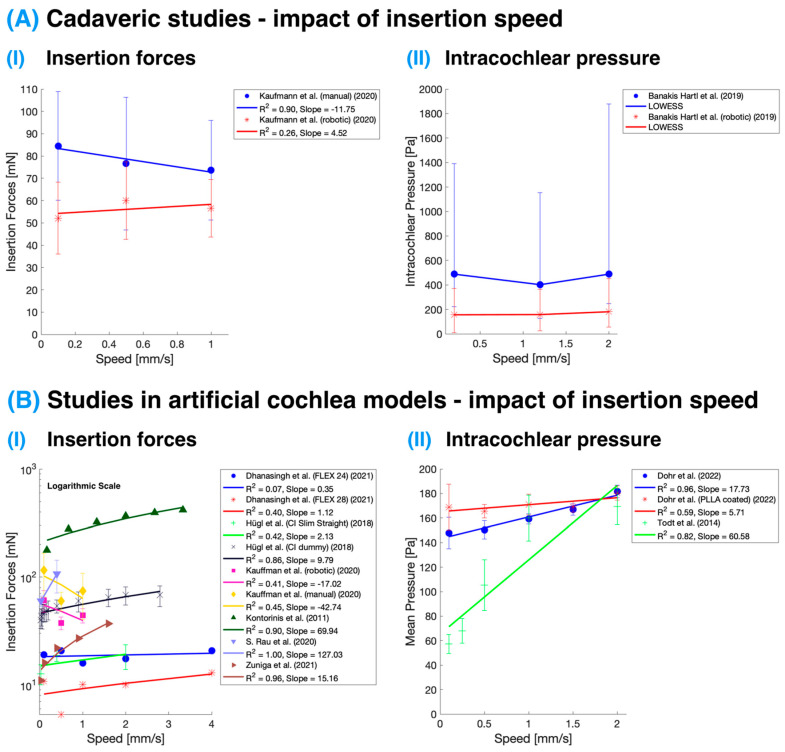
Impact of insertion speed on insertion forces and intracochlear pressure. (**A**) Studies were performed on cadaveric specimens [11,52]. The study of intracochlear pressure was evaluated using a nonparametric regression technique called Locally Weighted Scatterplot Smoothing (LOWESS). (**B**) Studies performed in artificial cochlea models [11,18,27,31,34,40,43,51,53]. The impact of insertion speed on insertion force is displayed in the logarithmic *y*-axis; hence, the linear regression lines appear curved. The color corresponds with the study, and the points and error bars represent mean and standard deviation, except in the case of cadaveric intracochlear pressure measurement, where these represent the median and interquartile range.

The analysis of the effect of insertion speed on cochlear implant IFs and IP revealed a trend found in studies performed in artificial models: higher insertion speeds are associated with increased IFs and higher IP (see Table 1 and Table 2 and Figure 2B). However, this effect was not confirmed in cadaveric studies (see Table 2 and Figure 2A), which seems to suggest these variables remain relatively stable. The variability in results between the cadaveric and artificial studies might be partly attributed to the larger number of studies in the artificial category compared to those conducted on cadaveric specimens. Among the latter, only two studies were conducted using fresh temporal bones; one focused on IFs, and the other on IP. Kaufmann et al. [11] report that insertion speed did not appear to have a substantial effect on the observed maximum forces in either artificial or cadaveric test specimens, likely due to the low-speed range tested. The chosen insertion speeds of 0.1, 0.5, and 1.0 mm/s represented the low-end and average typical reported manual insertion speeds, as well as constant robotics-assisted speeds below human limits. The lack of a strong correlation between insertion speed and insertion forces in this study could be attributed to the limited speed range examined.

**Table 1:** Summary of intracochlear pressure measurements in studies using artificial cochlea models. The primary measurements include mean pressure during CI insertion (measured in the scala tympani or in the lumen in cases where the implant was inserted into models with all scalae fused together), mean amplitude (calculated by averaging three to five of the largest changes), and peak frequency (indicative of the number of peaks over the course of the insertion). The term ‘Robotic?’ denotes the use of semi-automatic or automatic systems (e.g., stepper motor) during the insertion process. Term ‘Steering’ refers to the regulation or control exerted over the CI during insertion. Values are presented as (mean ± standard deviation) where data allowed. LW—lateral wall CI; PM—perimodiolar wall CI; N—number of insertions.

Author	Mean Pressure (Pa)	Amplitude (Pa)	Peak Frequency	Speed (mm/s)	Robotic	Steering	CI Type	CI Brand	N	Model
Dohr et al., 2022 [34]	(148 ± 13)	-	-	0.1	Y	N	LW	MED-EL	10	Artificial
	(150 ± 7)	-	-	0.5	Y	N	LW	MED-EL	10	Artificial
	(159 ± 4)	-	-	1	Y	N	LW	MED-EL	10	Artificial
	(167 ± 5)	-	-	1.5	Y	N	LW	MED-EL	10	Artificial
	(182 ± 5)	-	-	2	Y	N	LW	MED-EL	10	Artificial
	(169 ± 19)	-	-	0.1	Y	N	LW	MED-EL	10	Artificial
	(165 ± 5)	-	-	0.5	Y	N	LW	MED-EL	10	Artificial
	(171 ± 8)	-	-	1	Y	N	LW	MED-EL	10	Artificial
	(169 ± 4)	-	-	1.5	Y	N	LW	MED-EL	10	Artificial
	(180 ± 6)	-	-	2	Y	N	LW	MED-EL	10	Artificial
Mittmann et al., 2018 [19]	-	(14 ± 5)	(6.4 ± 0.4)	0.5	N	N	PM	HiFocus Midscalar AB	5	Artificial
	-	(8 ± 1)	(8.2 ± 0.2)	0.5	N	N	LW	LW prototype	5	Artificial
Mittmann et al., 2017 [35]	-	(35 ± 16)	(18 ± 2)	0.5	Y	N	LW	HiFocus 1J AB	3	Artificial
	-	(27 ± 8)	(14 ± 2)	0.5	Y	N	LW	HiFocus 1J AB	3	Artificial
Mittmann et al. (A) 2017 [36]	(115 ± 7)	(12 ± 9)	(12 ± 1)	0.48	N	N	LW	Slim Straight Cochlear	5	Artificial
	(149 ± 20)	(51 ± 9)	(12 ± 1)	0.48	N	N	PM	Nucleus 24 Cochlear	5	Artificial
Todt et al., 2017 [22]	(96 ± 19)	(24 ± 11)	(11 ± 2)	0.5	N	N	LW	HiFocus 1J AB	5	Artificial
	(109 ± 31)	(13 ± 13)	(4 ± 1)	0.5	N	N	PM	AB Helix	5	Artificial
	(80 ± 21)	(21 ± 13)	(13 ± 4)	0.5	N	N	PM	AB HF Midscalar	5	Artificial
	(51 ± 16)	(25 ± 39)	(11 ± 2)	0.5	N	N	LW	AB LW23	5	Artificial
Todt et al., 2014 [31]	(57 ± 8)	-	-	0.1	Y	N	LW	HiFocus 1J AB	3	Artificial
	(68 ± 10)	-	-	0.25	Y	N	LW	HiFocus 1J AB	3	Artificial
	(105 ± 21)	-	-	0.5	Y	N	LW	HiFocus 1J AB	3	Artificial
	(160 ± 19)	-	-	1	Y	N	LW	HiFocus 1J AB	3	Artificial
	(169 ± 15)	-	-	2	Y	N	LW	HiFocus 1J AB	3	Artificial
Todt et al., 2016 [30]	-	(87 ± 19)	(21 ± 2)	0.2	N	N	LW	HiFocus 1J AB	3	Artificial
	-	(25 ± 227)	(11 ± 1)	0.2	N	N	LW	HiFocus 1J AB	3	Artificial
	-	(8 ± 4)	(7 ± 1)	0.2	N	N	LW	HiFocus 1J AB	3	Artificial
	-	(20 ± 9)	(9 ± 2)	0.2	Y	N	LW	HiFocus 1J AB	3	Artificial
	-	(12 ± 5)	(8 ± 1)	0.2	Y	N	LW	HiFocus 1J AB	3	Artificial
	-	(4 ± 3)	(3 ± 1)	0.2	Y	N	LW	HiFocus 1J AB	3	Artificial
Todt et al. (A) 2016 [21]	-	(63 ± 8)	-	0.33	N	N	LW	HiFocus 1J AB	5	Artificial
	-	64 ± 4400)	-	0.33	N	N	LW	HiFocus 1J AB	5	Artificial
	-	(224 ± 60)	-	0.33	N	N	LW	HiFocus 1J AB	5	Artificial

**Table 2:** Overview of insertion force measurements in studies using cadaveric and artificial cochlea models. The CI insertion length is quantified either as angular insertion depth (in degrees) or as linear insertion distance (in millimeters). The term ‘Robotic?’ denotes the use of semi-automatic or automatic systems (e.g., stepper motor) during the insertion process. Term ‘Steering?’ refers to the regulation or control exerted over the CI during insertion. Values are presented as (mean ± standard deviation) where data allowed. LW—lateral wall CI; PM—perimodiolar wall CI; N—number of insertions.

Author	Force (mN)	Depth (deg/mm)	Speed (mm/s)	Robotic	Steering	CI Type	CI Brand	N	Model
Adunka et al., 2004 [13]	16 ± 4 to 218 ± 44	20 mm/300 deg	0.33 to 1.25	Y/N	N	LW	Various MED-EL	4 to 27	Artificial/ Cadaveric
Aebischer et al., 2022 [37]	9 ± 1 to 148 ± 32	20 mm/300 deg	0.33 to 1.25	Y	N	LW	Custom MED-EL	4 to 60	Artificial
Avci et al., 2017 [38]	29 ± 3	20 mm	1.25	Y	N	LW	Custom	4	Artificial
Bruns et al., 2020 [39]	17 ± 7	20 mm	0.33 to 1.25	Y	N	LW	MED-EL Flex	4 to 12	Artificial
De Seta et al., 2017 [10]	218 ± 44	22 mm	0.5	Y	N	LW	AB HiFocus 1J	4	Cadaveric
Dhanasingh et al., 2021 [40]	59 ± 24	20 mm	0.33 to 1.25	Y/N	N	LW	MED-EL Flex	6	Cadaveric
Hendricks et al., 2021 [41]	10 ± 2	20 mm	1.25	Y	N/Y	LW	Custom MED-EL	6 to 9	Artificial/ Cadaveric
Hrncirik et al., 2022 [23]	5 ± 1 to 15 ± 9	20 mm/300 deg	0.4 to 1.25	Y	N/Y	LW	Custom MED-EL	9 to 12	Artificial
Hügl et al., 2019 [42]	33 ± 4 to 108 ± 49	20 mm	0.33 to 2	Y	N	LW	Various MED-EL	9 to 35	Artificial
Hügl et al. (A) 2018 [43]	40 ± 7 to 418 ± 8	17 mm	0.03 to 3.33	Y	N	LW	Custom	12 to 36	Artificial/ Cadaveric
Kauffman et al., 2020 [11]	46 ± 13	17 mm	0.06 to 0.8	Y	N	LW	Custom	12	Artificial
Kontorinis et al., 2011 [27]	47 ± 8	17 mm	0.06 to 0.8	Y	N	LW	Custom	12	Artificial
Leon et al., 2017 [14]	47 ± 9	17 mm	0.11 to 0.15	Y	N	LW	Custom	12	Artificial
Majdani et al., 2010 [15]	48 ± 9	17 mm	0.2 to 0.4	Y	N	LW	Custom	12	Artificial
Miroir et al., 2012 [44]	50 ± 10	17 mm	0.2 to 0.4	Y	N	LW	Custom	12	Artificial
Mirsalehi et al., 2017 [45]	54 ± 8	17 mm	0.4 to 0.9	Y	N	LW	Custom	12	Artificial
Nguyen et al., 2015 [29]	60 ± 13 to 479 ± 116	17 mm	0.9 to 2	Y/N	N	LW	Custom	8 to 39	Artificial/ Cadaveric
Radeloff et al., 2009 [46]	255 ± 75	25 mm	0.6	Y	N	LW	AB HiFocus 1J	39	Cadaveric
Riojas et al., 2021 [47]	62 ± 17	20 mm/300 deg	0.5	Y	N	LW	Digisonic SP	8	Cadaveric
Rohani et al., 2014 [48]	21 ± 3	20 mm	0.5	Y	N	LW	Custom	15	Cadaveric
S. Rau et al., 2020 [18]	479 ± 116	20 mm	0.5	Y	N	LW	MED-EL C40+	8	Artificial
Schurzig et al., 2010 [49]	257 ± 89	17 mm	0.2 to 0.4	Y	N	LW	Custom	12	Artificial
Smetak et al., 2023 [50]	9.71 to 9.97	17 mm	0.4 to 1.6	N	N	LW	MED-EL	4	Artificial
Wrzeszcz et al., 2015 [28]	75 ± 20 to 418 ± 8	17 mm to 25 mm	0.03 to 3.33	Y	N	LW/PM	Custom	9 to 36	Artificial/ Cadaveric
Zuniga et al., 2021 [51]	256 ± 61 to 418 ± 8	25 mm	0.6 to 3.33	N	N	LW	AB HiFocus 1J	39	Cadaveric

### 3.2. Distance and Angular Depth of Insertion

**Figure 3:**
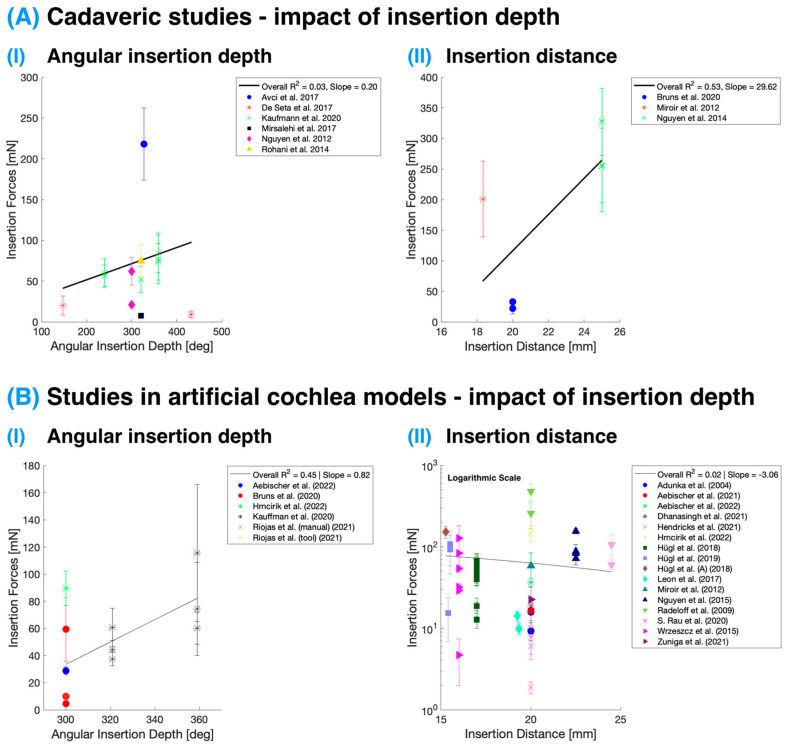
Impact of insertion depth on the IFs measured in cadaveric specimens (**A**) [10,11,38,39,44,45,48,57] and in artificial cochlea models (**B**) [11,13,14,18,23,28,29,37,39,40,41,42,43,44,46,47,51,53,58] with angular insertion depth in degrees and insertion distance in mm. (**B-II**) has *y*-axis with a logarithmic scale. The color corresponds with the study, and the points and error bars represent the mean and standard deviation.

Figure 3 presents the effects of angular insertion depth and insertion distance on IFs in studies conducted on both cadaveric specimens and artificial cochlea models. The IFs displayed are the maximum forces reported in these studies at the specified depths/distances. It is essential to note that intracochlear pressure measurements are not included in this analysis as pressure typically equalizes rapidly during insertion. Consequently, changes in pressure with the final implant position within the cochlea are minimal, and such data are often not included in studies. Nevertheless, the rate of pressure equalization is highly dependent on the size of the round window or cochleostomy opening [21,54,55], as discussed in the previous section. This factor could lead to slower pressure equalization during insertion and, thus, observable IP changes at varying insertion depths. Following this, Ordonez et al. [56] reported possible higher pressure levels with deeper insertions attributed to the decreasing volume of scala tympani at deeper levels.

### 3.3. Controlled Robotic Insertion

**Figure 4:**
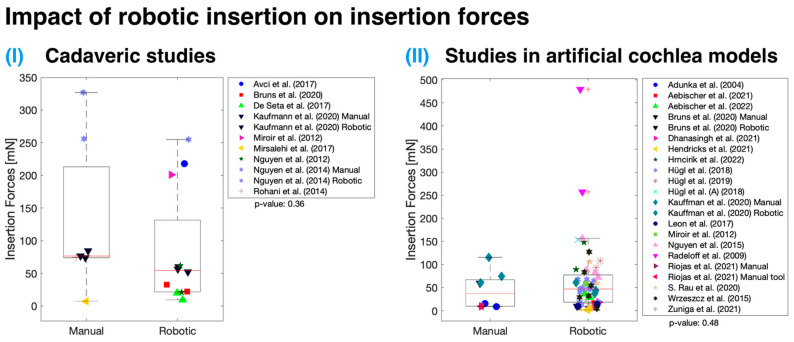
Impact of robotic insertion on mean maximal IFs measured in cadaveric specimens (**I**) [10,11,26,38,39,44,45,48,57] and artificial cochlea models (**II**) [11,13,14,18,23,28,29,37,39,40,41,42,43,44,46,47,51,53,58]. Unweighted two-sample *t*-tests were used for both diagrams with no significant difference found (box charts not weighted). Red line—median; black box—interquartile range; red crosses—outliers.

Both cadaveric specimen and artificial cochlea model measurements indicate no statistically significant difference (*p* > 0.05) in mean maximal IFs when employing controlled robotic assistance for insertion (Figure 4). The analysis utilized an unweighted *t*-test as the number of studies for each group (manual or robotic) was not equal. It is essential to note that the data are plotted regardless of any other variables that might have contributed to the lack of significant difference between IFs measured using manually and automatically inserted cochlear implants, though it may be that no true difference exists. Only three studies [11,39,57] have investigated the use of robotic insertion and compared it to standard manual insertion performed by a surgeon. Among these, only Kaufmann et al. had an adequate sample size [11]. According to their data, there was a significant reduction (*p* < 0.001) in IFs with robotic insertions when compared to manual insertions, measured in both cadaveric specimens and artificial cochlea models.

**Figure 5:**
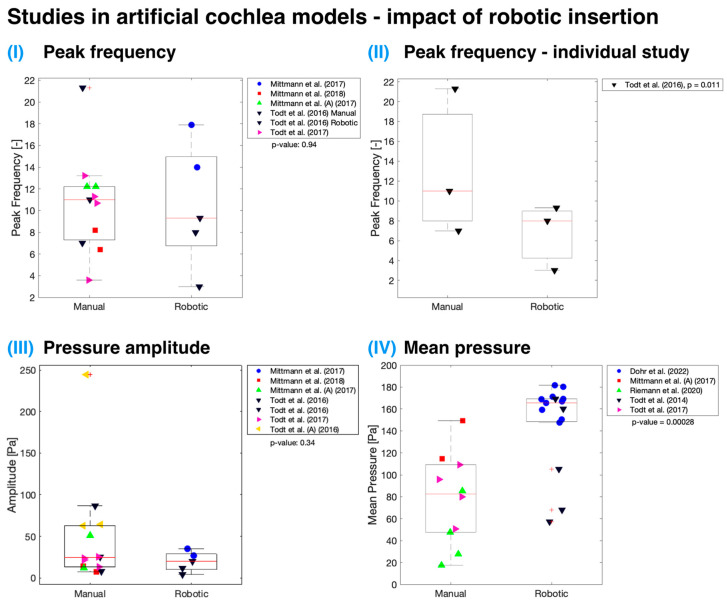
Impact of robotic insertion on pressure measurements in artificial cochlea models [19,20,22,30,31,34,35,36]. Unweighted two-sample *t*-tests were used for (**I**,**III**,**IV**), with significant differences found in mean pressure measurements (box charts not weighted, *p* < 0.001). A weighted *t*-test was used for (**II**), highlighting a significant difference between manual and robotic insertion (*p* = 0.011). Red line—median; black box—interquartile range; red crosses—outliers.

Figure 5 presents studies conducted on artificial cochlea models that demonstrate various pressure parameters, including frequency of pressure peaks, mean pressure amplitudes, and mean pressure, which could be affected by robotic insertion. Robotically assisted insertion was not found to be significant (*p* > 0.05) for all parameters except for mean pressure (*p* < 0.001). One explanation for this may be that a large proportion of the data on this variable come from the work of Dohr et al., which used a linear artificial scala tympani model, which might have contributed to the higher observed pressures [34]. Additionally, the authors focused on measuring maximum pressure during the insertion process, which might differ from the mean pressure. A broader issue in pressure-measurement studies in the cochlea is the varying definitions of variables, contributing to inconsistencies across findings.

A single study by Todt et al. directly contrasted robotic and manual insertion techniques from the standpoint of sudden pressure changes [30]. They observed a significant difference for both the number of pressure peaks (*p* = 0.011 when all manual and robotic insertion data points were compared together; see Figure 5II) and the mean amplitude (*p* = 0.027) size that arose during implant insertion into the artificial cochlea model. They noted that manual insertion introduced hand tremors, which increased the pressure peak frequency and amplitude. The tremor and sudden movements during insertion could be minimized by supporting the surgeon’s hand and by using semi-automated or fully automatic insertion systems [30]. Nonetheless, implant volume is an important factor, as larger implants displace greater amounts of fluid within the cochlea, resulting in increased pressure [22,54]. This effect is particularly evident when comparing lateral wall and perimodiolar wall implants [59].

### 3.4. Distribution of Insertion Trauma

**Figure 6:**
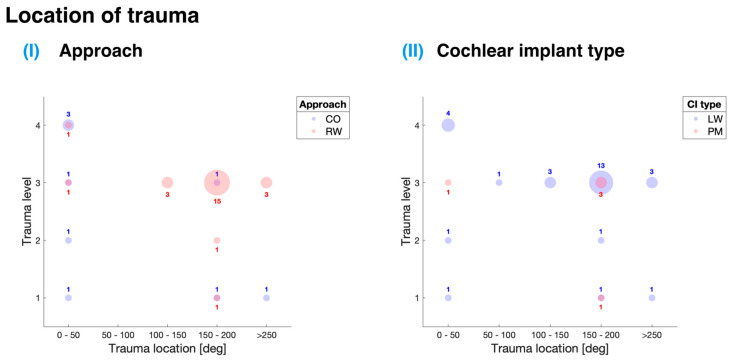
Distribution of trauma occurrences by angular insertion depth, surgical approach, and cochlear implant type in human cadaveric cochlear implantation studies: (**I**) comparison of cochleostomy (CO) and round window (RW) approaches. (**II**) evaluation of lateral wall (LW) and perimodiolar (PM) cochlear implants. Trauma locations are categorized by angular insertion depth in degrees: 0–50; 50–100; 100–150; 150–200; 200–250; above 250 degrees. Trauma levels follow the Eshraghi et al. scale [12]. Bubble size and accompanying number indicate the number of trauma occurrences.

Figure 6 offers an in-depth analysis of the data gathered from the selected studies, presenting a depiction of the association between the location and level of trauma in relation to different cochlear implant insertion approaches and implant types. This scoping review paid particular attention to two main insertion approaches: entry through the RW and CO. Table 3 shows that the majority of the analyzed papers (five out of six) employed the RW approach for cochlear implant insertion, which suggests a preference for this method within the studies evaluated.

For the RW approach, the most commonly observed insertion trauma was level 3 (see Figure 6I), which corresponds to the rupture of the basilar membrane as defined by the Eshraghi et al. scale [12]. This trauma level can have significant implications for the patients, as it may result in reduced hearing performance and hinder the overall effectiveness of the cochlear implant. The preponderance of trauma instances for this approach was found at 150° to 200° of angular insertion depth, amounting to 15 occurrences. Furthermore, there were three instances, each at 100° to 150° and more than 250° of angular insertion depth. The distribution of these instances may indicate potential areas for improvement in the RW approach to minimize the occurrence of insertion trauma.

## 4. Discussion

This scoping review attempts to provide a comprehensive analysis of the factors affecting IFs and IP during cochlear implantation, with particular emphasis on insertion depth, cochlear implant design, and robotic assistance. Alongside these considerations, the review also offered key insights into the occurrence and distribution of cochlear trauma in relation to different cochlear implant insertion approaches and implant types.

### 4.1. Implant Design (Lateral Wall vs. Perimodiolar)

The majority of studies in this review focused on lateral wall cochlear implants, which exhibit a consistent IF profile. A parallel trend was found in the assessment of cochlear trauma, with LW implants being the predominant type in the analyzed studies. The highest occurrence of trauma was found to be level 3 on the Eshraghi et al. scale [12], corresponding to the rupture of the basilar membrane. Although the existing data do not allow for a definitive comparison between LW and PM cochlear implants, these findings underscore the significance of implant design and surgical approach in determining both IFs and trauma levels.

Perimodiolar implants were not included in the IF analysis due to their distinct insertion profile and because no study directly contrasted the IF profiles of perimodiolar and lateral wall cochlear implants. Additionally, no studies in the trauma review directly compared LW and PM cochlear implants within the same investigation. Future research should address these gaps by investigating the differences in IF and trauma profiles between these implant types to inform clinicians about the most appropriate implant type for specific patient cases.

A few studies not included in this review investigated the synergistic impact of implant design and insertion approach. Wanna et al. conducted a comprehensive study examining 100 postlingually deafened adult patients with a total of 116 implants comprising both LW and PM designs [60]. The surgical approaches employed were CO, extended round window (ERW), and RW. The study found that LW electrodes had a higher rate of complete ST insertion compared to PM designs (89% vs. 58%). Both ERW and RW procedures resulted in higher rates of complete ST insertion compared to CO procedures. A statistically significant difference in consonant-nucleus-consonant word recognition was identified with the group with electrode placement entirely within the ST exhibiting higher mean consonant-nucleus-consonant scores than the group with placement outside the ST (48.9% vs. 36.1%; *p* < 0.045). Interestingly, no statistically significant differences were observed among the three device manufacturers regarding the rate of complete ST-electrode insertion or audiometric performance when comparing LW electrodes. It should be noted that PM electrode designs have evolved with time, and modern PM electrodes may potentially be less traumatic, but this remains to be proven.

Jwair et al. conducted a systematic review and meta-analysis to investigate scalar translocation in cochlear implantation and its relationship with speech perception scores and residual hearing [61]. The authors discovered a significantly lower scalar translocation rate for LW electrode arrays compared to PM electrode arrays, which is consistent with Wanna et al.’s findings [60]. Furthermore, similar to our results, the study found that translocations predominantly occurred at an angular insertion depth of approximately 180°, primarily with PM arrays. The researchers posited that the primary reason for the occurrence of translocations at this specific depth could be attributed to a steep decrease in the dimensions of the scala tympani and the cochlear hook region, situated at the base of the cochlea, exhibiting a complex and heterogeneous shape, which could increase the likelihood of trauma. Lastly, increased intracochlear friction might also play a role in the occurrence of translocations at this depth.

The potential influence of implant type on the level and location of trauma warrants further consideration. Future research endeavors could benefit from a more in-depth exploration of the effects of implant design and materials on insertion trauma, as well as an examination of the possible synergistic impact resulting from the combination of insertion approach and implant type. By investigating these factors, researchers may be able to identify crucial elements that contribute to reduced cochlear trauma and improved patient outcomes in cochlear implantation procedures.

### 4.2. Surgical Approach (Cochleostomy vs. Round Window)

In contrast to the RW approach, the CO approach exhibited a higher number of trauma instances at 0° to 50° of angular insertion depth. This finding is particularly intriguing, as these instances might be ascribed to the trauma stemming from the location and angle of the cochleostomy site rather than being explicitly related to the electrode carrier. This observation raises questions about the surgical technique employed in the CO approach and its potential to inadvertently cause trauma.

The group of Adunka et al. emphasized that in four bone samples, grade 2 to 4 trauma was only discernible in the region of the cochleostomy [13]. This discovery suggests that the observed trauma is more likely a result of the surgical procedure itself, as opposed to being directly induced by the electrode array. Consequently, it raises concerns about the need to refine the CO approach to mitigate the risks associated with the surgical process.

### 4.3. Angular Insertion Depth and Intracochlear Trauma

The trauma locations identified in this work are in good accordance with the findings of Ishiyama et al., who performed a histopathological study of human temporal bones from CI patients who exhibited intracochlear injury related to translocation (trauma level 4) [62]. The authors found that nearly all cases of electrode translocation or migration occurred near the same angular insertion depth, which was at or near 180° of angular insertion depth, with the junction between the descending and ascending basal turn of the cochlea appearing to be the area susceptible to translocation from scala tympani to scala vestibuli. The authors suggest that the triggering of osteoneogenesis from the site of cochleostomy due to endosteal damage may play a role in the increased severity of intracochlear damage when translocation occurs in the setting of cochleostomy. They recommend using the round window approach with careful drilling of the operculum for the placement of CI to minimize intracochlear trauma.

Similarly to the Ishiyama et al. study, the group of Fayad et al. also conducted a histopathological study which investigated the CI insertion damage, the relationship between dendrite and spiral ganglion cell populations, and the structures stimulated by CI [63]. The study revealed that the primary site of trauma was the anterior part of the basal turn, where the electrode made contact with the outer wall of the cochlea before bending towards the modiolus. This led to disruption of the spiral ligament and stria vascularis, resorption of the organ of Corti, ossification from the damaged endosteum, fracture of the osseous spiral lamina, and degeneration of dendrites. The study also found that the structures stimulated by cochlear implants are primarily the spiral ganglion cells or axons and not the dendrites. Furthermore, the research suggested that response to stimulation could occur with as little as 10% of the normal population of ganglion cells, highlighting that fewer ganglion cells than previously thought might be necessary for successful stimulation.

Figure 6II and Table 3 demonstrate that LW cochlear implants were predominantly used in the analyzed studies. The highest occurrence of trauma for LW implants was observed at level 3, which corresponds to the rupture of the basilar membrane, a critical structure for hearing. This trauma was primarily found at approximately 150° to 200° of angular insertion depth. Additionally, a considerable level of trauma (level 4) was observed at 0° to 50° insertion depths, corresponding to the translocation of the CI from scala tympani to scala vestibuli. The study by Adunka et al. indicated that this trauma is likely attributable to the surgical procedure rather than the specific cochlear implant type [13].

Regarding PM CI insertion, Figure 6II indicates five occurrences of trauma, primarily at 150° to 200° insertion depths and level 3 insertion trauma. These findings were derived from a study conducted by Briggs et al., which introduced a novel thin perimodiolar prototype electrode array [9]. The study employed a multi-center collaborative approach with a large cohort of surgeons, emphasizing that the cochlear implant’s size and shape are crucial factors in minimizing trauma during implantation. A thin and flexible electrode array was shown to reduce the risk of intracochlear trauma, highlighting the importance of considering size and shape in cochlear implant design to optimize patient outcomes and minimize surgical complications.

## 5. Limitations and Future Research

It is important to acknowledge the limitations of the selected studies for trauma distribution, as none directly compared LW and PM cochlear implants within the same investigation. As a result, definitive conclusions regarding the superiority of one implant type over the other cannot be drawn based on the existing data. Nevertheless, the findings underscore the significance of implant design and surgical approach in reducing cochlear trauma and enhancing patient outcomes.

While the collective analysis of the studies included in this review presented variability in the relationship between IFs and different metrics of insertion (both distance and angular depth) due to a myriad of variables across research groups, the study by Kaufmann et al. [11] distinctly demonstrated the correlation between IFs and angular insertion depth. Their data showed that with an increase in angular insertion depth, there was a corresponding increase in IFs. This insight underscores the importance of evaluating IFs specifically against angular insertion depth for a more nuanced and meaningful comparison within the literature.

The reviewed studies exhibited considerable variability in methodology, implant type, and measurement techniques. Future research should aim to develop more standardized and accurate models that closely mimic the in vivo situation, and a standardization of methods and reporting across studies would facilitate better comparisons. This holds true for both IF and cochlear trauma research, contributing to a more robust understanding of the factors influencing IFs, IP, and cochlear trauma during cochlear implantation.

In terms of cochlear trauma, the findings highlight that the RW approach is the preferred insertion method among the studies analyzed, with the majority of trauma instances occurring at an angular insertion depth of 150° to 200°. In contrast, the CO approach showed a higher incidence of trauma at 0° to 50° of angular insertion depth. This raises questions about the surgical technique used in the CO approach and its potential to inadvertently cause trauma.

Regarding robotic assistance, the analysis revealed no statistically significant difference in mean maximal IFs between manually and robotically inserted cochlear implants. However, robotic assistance may offer potential benefits such as reduced hand tremor and more controlled insertion. The impact of robotic assistance on cochlear trauma remains an open question, and future studies should address this by focusing on its potential impact on patient outcomes and residual hearing preservation.

There are a number of other areas that could not be addressed as they were not explored in any of the primary studies. These include methods of securing the electrode following insertion and the impact of packing the round window or cochleostomy. These merit further exploration.

## 6. Conclusions

In conclusion, this review has emphasized the importance of insertion depth, cochlear implant design, and robotic assistance in determining IFs and IP during cochlear implantation, as well as in understanding the occurrence and distribution of cochlear trauma. Future research should focus on addressing the identified limitations and knowledge gaps to optimize cochlear implant surgery, improve patient outcomes, and preserve residual hearing. Through this endeavor, researchers and clinicians can collaborate to improve the overall effectiveness of cochlear implant procedures and enhance the quality of life for patients with hearing loss.

## Figures and Tables

**Table 3:** Summary of trauma incidences related to cochlear implantation, detailing the trauma location (in degrees of angular insertion depth) and level (according to Eshraghi et al.) [12], the surgical approach and implant type used, and the cochlear implant brand, as reported in various literature studies. CO—cochleostomy; RW—round window; LW—lateral wall CI; PM—perimodiolar wall CI.

Author	Trauma Location	Trauma Level	Approach	CI Type	CI Brand
Adunka et al., 2004 [13]	0–50	4	CO	LW	C40+ Flex MED-EL
	0–50	4	CO	LW	C40+ Flex MED-EL
	0–50	4	CO	LW	C40+ Flex MED-EL
	0–50	2	CO	LW	C40+ Flex MED-EL
	0–50	1	CO	LW	C40+ Flex MED-EL
	250	1	CO	LW	C40+ Flex MED-EL
Kaufmann et al., 2020 [11]	0–50	4	RW	LW	Unknown
	150–200	1	RW	LW	Unknown
	150–200	3	RW	LW	Unknown
Briggs et al., 2011 [9]	0–50	3	CO	PM	Custom
	150–200	1	CO	PM	Custom
	150–200	3	CO	PM	Custom
	150–200	3	RW	PM	Custom
	150–200	3	RW	PM	Custom
De Seta et al., 2017 [10]	150–200	3	RW	LW	Flex 28 MED-EL
	150–200	3	RW	LW	Flex 28 MED-EL
	150–200	3	RW	LW	Flex 28 MED-EL
	150–200	3	RW	LW	Flex 28 MED-EL
	150–200	3	RW	LW	Flex 28 MED-EL
	150–200	2	RW	LW	Flex 28 MED-EL
Mirsalehi et al., 2017 [45]	100–150	3	RW	LW	Slim Straight Cochlear
	150–200	3	RW	LW	Slim Straight Cochlear
	100–150	3	RW	LW	Slim Straight Cochlear
	100–150	3	RW	LW	Slim Straight Cochlear
Nguyen et al., 2012 [26]	50–100	3	RW	LW	Digisonic SP
	150–200	3	RW	LW	Digisonic SP
	150–200	3	RW	LW	Digisonic SP
	150–200	3	RW	LW	Digisonic SP
	150–200	3	RW	LW	Custom
	150–200	3	RW	LW	Custom
	150–200	3	RW	LW	Custom
	<250	3	RW	LW	Custom
	<250	3	RW	LW	Custom
	<250	3	RW	LW	Custom

## Data Availability

Data from this study are available from the authors. Data from primary studies should be requested from the authors of those works.
